# Home-Based, Telematic Gradual Exercise for Permanent Colostomy Patients: Protocol for a Randomized Controlled Trial

**DOI:** 10.3390/healthcare13212742

**Published:** 2025-10-29

**Authors:** Ángel Antequera-Antequera, Geraldine Valenza-Peña, Julia Raya-Benítez, Alba Navas-Otero, Marie Carmen Valenza, Andrés Calvache-Mateo, Irene Cabrera-Martos

**Affiliations:** 1 Department of Physiotherapy, Faculty of Health Sciences, University of Granada, Av. De la Ilustración, 60, 18016 Granada, Spain; antequerafisio@gmail.com (Á.A.-A.); geraldinevalenza@ugr.es (G.V.-P.); albanavas@ugr.es (A.N.-O.); cvalenza@ugr.es (M.C.V.); irenecm@ugr.es (I.C.-M.); 2 Department of Nursing, Faculty of Health Sciences, University of Granada, 18016 Granada, Spain; juliarb@ugr.es

**Keywords:** permanent colostomy, exercise therapy, home-based intervention, quality of life, kinesiophobia, self-efficacy, randomized controlled trial

## Abstract

**Background/Objectives:** Permanent colostomy requires significant physical and psychological adaptation. Patients often experience reduced physical activity, impaired quality of life, and fear of movement. Current exercise recommendations are inconsistent, and no consensus exists on safe return to activity. This study aims to evaluate the effect of a 12-week home-based graded exercise programme on physical activity, quality of life, kinesiophobia, exercise capacity, and self-efficacy in patients with permanent colostomies. **Methods:** This randomized controlled trial will recruit 51 adults with permanent colostomies, beginning six weeks post-surgery. Participants will be randomized (1:1) to an intervention or control group. The intervention group will receive a 12-week home-based exercise programme including patient education, resistance and core training, and progressive aerobic walking. The control group will receive standard medical care and an informational leaflet. Primary outcomes include physical activity (steps/day), quality of life (Stoma-QoL), kinesiophobia (Tampa Scale), exercise capacity (6-Minute Walk Test), and self-efficacy (General Self-Efficacy Questionnaire). Follow-up will be conducted at baseline, post-intervention, and six months. Data will be analyzed using intention-to-treat principles with a significance threshold of *p* < 0.05. **Conclusions:** This trial will be the first to assess the effects of a structured, home-based graded exercise programme in individuals with permanent colostomies. The findings are expected to provide evidence on the efficacy of exercise for improving physical and psychological outcomes in this population and to inform clinical guidelines for safe, individualized activity resumption.

## 1. Introduction

A colostomy is a surgical procedure performed to divert intestinal transit when anatomical or functional abnormalities prevent normal fecal evacuation through the anal canal. It creates an opening in the abdominal wall through which waste is expelled into an external pouch [1,2].

Colostomies represent a primary therapeutic option for conditions such as colorectal cancer, diverticulitis, severe trauma, inflammatory bowel diseases, and congenital disorders. Depending on the underlying cause, colostomies may be temporary or permanent. Permanent colostomies, in particular, require substantial adaptation, as they affect gastrointestinal function, body image and daily activities [3].

In the United States, it is estimated that nearly one million individuals currently live with an ostomy, with 100,000 to 130,000 new procedures performed each year [4]. Across the European Union, it is estimated that 725,000 to 1 million individuals live with an ostomy, a figure expected to rise in parallel with the increasing prevalence of inflammatory bowel disease and other gastrointestinal disorders [5]. This condition may lead to complications that adversely affect quality of life, often resulting in physical, psychological and social challenges [4,6,7,8,9,10].

Stoma-related issues, such as bleeding, necrosis, prolapse and hernias, are common and frequently accompanied by sexual dysfunction, fatigue and bowel irregularities [1,11,12,13]. These impairments are closely linked to psychological distress, including depression, anxiety [14], disturbances in body image [15] and decreased physical activity [16,17].

Russell et al. investigated the attitudes and perceptions of 2631 ostomy patients regarding exercise, reporting a prevalent fear of physical activity, low activity levels, limited knowledge, and the perception of parastomal hernia risk as a major barrier to participation in exercise programmes [17]. Interventions in this population have primarily targeted hernia prevention [18], improvements in quality of life [19,20,21,22,23], pain reduction, and mental health support [22,24,25]. However, there is currently no consensus regarding the safe resumption of physical activity following colostomy, highlighting the need for gradual, individualized exercise programmes that educate patients and promote safe and progressive activity.

The selection of study variables was based on the multifactorial nature of adaptation following colostomy. Physical activity was included as a primary indicator of behavioural change and global recovery, since reduced activity levels have been consistently linked to postoperative complications and poorer health outcomes [26,27,28]. Quality of life represents a central construct integrating physical, psychological, and social dimensions, which are often impaired in this population. Kinesiophobia, or fear of movement, was incorporated because it constitutes a psychological barrier to physical rehabilitation and has been associated with decreased participation and delayed functional recovery in various clinical populations [29,30]. Exercise capacity, measured through the Six-Minute Walk Test, provides an objective index of functional performance and tolerance to physical effort [31]. Finally, self-efficacy was selected as it reflects patients’ confidence in managing their condition and performing physical activity safely, a critical determinant of adherence and long-term lifestyle modification [32]. Together, these outcomes provide a comprehensive assessment of both physical and psychological domains relevant to recovery after permanent colostomy.

Given the heterogeneity of exercise recommendations for permanent colostomy patients, personalized home-based programmes are required to reduce barriers and improve adherence. The present study aims to evaluate the effect of a home-based gradual exercise programme on physical activity, quality of life, kinesiophobia, exercise capacity, and self-efficacy in individuals with permanent colostomies. A six-month follow-up will also be conducted to assess patient adherence and programme satisfaction. We hypothesize that participants who complete the 12-week home-based gradual exercise programme will demonstrate greater improvements in physical activity levels, quality of life, kinesiophobia, exercise capacity, and self-efficacy compared with those receiving standard medical care.

## 2. Materials and Methods

### 2.1. Design

The study is designed as a randomized clinical trial to analyze the effect of a home-based gradual exercise programme in patients with a permanent colostomy, commencing six weeks after surgery. The research assistant responsible for data collection will be blinded to the study hypothesis and patient group allocation. Due to the nature of the intervention, participant blinding will not be feasible. The study protocol will be registered at Clinicaltrials.gov: DF0103UG.

### 2.2. Ethical Considerations

The study protocol will be approved by the local Research Ethics Committee. The trial will be conducted in accordance with the 1975 Declaration of Helsinki [33], as revised in 2024, and the Consolidated Standards of Reporting Trials (CONSORT) [34]. Written informed consent will be obtained from all participants after a detailed explanation of the study procedures.

### 2.3. Setting

This study will be conducted at Virgen de las Nieves Hospital in Granada (Spain). Participants will be recruited from the general digestive surgery department.

### 2.4. Participants

Patients over 18 years of age who have undergone surgical treatment resulting in a permanent colostomy will be eligible. A colostomy is a surgical opening of the large intestine (colon) through the abdominal wall, providing a new pathway for waste elimination. A colostomy is considered permanent when restoration of intestinal continuity is not possible or not planned [1,3].

Based on a review of current literature, six weeks postoperatively is considered an appropriate timeframe for patients to adjust to their new condition and home environment, making this period suitable for initiating a home-based exercise intervention [4].

Exclusion criteria will include the following: pulmonary, cardiac, neurological, vascular, or orthopedic conditions limiting participation; cognitive impairment preventing comprehension of the intervention; ostomy-related complications; and refusal to provide informed consent.

### 2.5. Randomization

Eligibility will be assessed by an impartial professional uninvolved in the randomization process. Randomization will be performed by an independent researcher who will not participate in patient assessment or treatment. Patients will be assigned sequentially to groups using a computer-generated randomization sequence (https://www.randomizer.org) with a 1:1 allocation ratio. The randomization sequence will be securely maintained off-site. Group allocation will be communicated to the recruiter via email, and assessments will be carried out by a physiotherapist blinded to group allocation.

### 2.6. Intervention

The experimental group will participate in a 12-week home-based programme. This programme will include patient education on the benefits of physical exercise and guidance on how to perform it safely with a colostomy, resistance training targeting major muscle groups, core-strengthening exercises, and aerobic walking exercise. The Template for Intervention Description and Replication (TLDieR) checklist [35] will be followed. The control group will receive standard medical care along with an informational leaflet on colostomy management and the main barriers to physical activity they may encounter.

#### 2.6.1. Experimental Group

Participants assigned to the intervention group will undergo a 12-week home-based graded exercise programme, structured as follows:

Initial Session

The initial session will be conducted individually to inform the patient about the benefits of exercise and how to perform it safely with a colostomy. Questions will be addressed, and the structure of the intervention and data collection will be explained.

Week 1–4: Introduction and Supervised Sessions

Participants will complete one 15 min weekly supervised online session, including warm-up, resistance training, core training (based on Rune et al., 2020 recommendations) [36] and cool-down. Resistance exercise training will target major muscle groups involved in daily activities, including lunges, leg lifts, bridges, side bridges, upper limb raises, sit-to-stand and step-ups. Participants will also perform a second weekly self-paced walking session, starting at five minutes, progressively increasing by five minutes per week to a target of 45–60 min.

Week 5–8: Progressive Training

Participants will complete one supervised session, one additional session repeating the same exercises with physiotherapist support if needed and one walking session.

Week 9–12: Consolidation and Independent Sessions

Participants will perform two core and resistance exercise sessions and two walking sessions weekly.

Participants will record perceived exertion, pain, and discomfort after each session in a training diary, which will be reviewed weekly by the physiotherapist. Exercise intensity will be individually adjusted and monitored using the Borg Rating of Perceived Exertion (RPE) scale, targeting a moderate intensity level (12–14 on the 6–20 scale). The use of the RPE scale ensures that exercise load is safely tailored to each participant’s tolerance, minimizing the risk of adverse effects.

Supervised sessions will be conducted via a secure video-conferencing platform (Google Meet^®^), ensuring real-time interaction between the physiotherapist and the patient. Each session will include live demonstrations, performance feedback, and clarification of patient questions. Adherence and technical feasibility will be recorded.

Participants will receive both written and verbal instructions on how to access and use the Google Meet^®^ platform prior to the first supervised session. A brief test connection will be performed to ensure appropriate technical setup. All participants will maintain an exercise diary, available in both paper and digital format, to record session completion, perceived exertion, and any discomfort or adverse events. The physiotherapist will review these logs weekly via telematic communication and provide individualized feedback and motivational reinforcement. Additionally, participants will be able to contact the research team via an institutional email address for technical assistance or clarification during the intervention period. All telematic interactions will be conducted in compliance with institutional data protection and confidentiality policies.

#### 2.6.2. Control Group

The control group will receive standard medical care and an informational leaflet covering colostomy management and common barriers to physical activity. No supervised exercise intervention will be provided.

### 2.7. Outcomes

Patients will be contacted during follow-up visits conducted by the nursing service. Patients will be informed of the study, and if they agree to participate, a face-to-face appointment will be arranged. The initial evaluation will be performed once informed consent has been obtained. Data collection will include anthropometric variables (age, educational level, weight, height, marital status, employment status, and educational background), comorbidities assessed using the Charlson Comorbidity Index [37] and relevant clinical history data (medical history, indication for surgery, and regular medication). Participants’ perception of exercise will be measured using the Exercise Benefits/Barriers Scale [38].

#### 2.7.1. Primary Outcomes

Physical activity levels will be assessed via daily steps and energy expenditure, measured using a uniaxial accelerometer-based pedometer [39]. Participants will receive verbal and written instructions on its use. The device will be positioned at the left anterior superior iliac spine using a belt. Participants will be instructed to wear the pedometer throughout the day, except during sleep or bathing. For analysis, the mean values from the last seven consecutive days will be used. This device has demonstrated adequate reliability and validity in clinical and older populations [40,41]. The uniaxial accelerometer selected for this research has demonstrated adequate validity and reliability for step counting and energy expenditure estimation in clinical and older populations. Its simplicity and ease of use are advantageous for promoting adherence and minimizing technical difficulties in a home-based setting.

Quality of life will be assessed using the Stoma Quality of Life Questionnaire (Stoma-QoL). This disease-specific instrument has demonstrated strong reliability and validity, with good internal consistency and construct validity, and was developed to assess the physical, psychological, and social impact of living with a temporary or permanent stoma [42]. The questionnaire consists of 20 items covering domains such as social and leisure limitations, body image, physical activity, emotional wellbeing and stoma care. Each item is rated on a 4-point Likert scale ranging from “never” to “always”. Scores are transformed to a 0–100 scale, with higher values indicating better perceived quality of life. The Stoma-QoL has been validated in multiple languages, including Spanish, and demonstrates adequate reliability, making it suitable for both clinical and research settings [43,44].

Kinesiophobia is defined as an excessive, irrational and debilitating fear of movement, based on the perceived vulnerability to pain or reinjury [45]. It will be assessed using the 11-item version of the Tampa Scale of Kinesiophobia (TSK), a self-administered questionnaire widely employed to evaluate fear of movement or injury across diverse clinical populations. Each statement is rated on a 4-point Likert scale (1–4), yielding a total score ranging from 11 to 44, with higher scores indicating a greater level of kinesiophobia. The TSK has demonstrated strong reliability and validity, and a Spanish version is available. It shows adequate internal consistency and test–retest reliability, with a Cronbach’s alpha coefficient of 0.79 [46,47].

The Six Minute Walk Test (6MWT) is a functional test used to assess submaximal aerobic capacity and an individual’s functional status [48]. It is a simple, easy-to-administer test that measures the distance a person can walk along a flat, straight 30 m course over a period of six minutes. Heart rate, oxygen saturation, dyspnoea, and lower limb fatigue are monitored before and after the test. The 6MWT has demonstrated excellent reliability and validity and is widely used for the assessment and monitoring of chronic conditions [49], including cardiovascular and respiratory diseases, musculoskeletal conditions [50], cancer [31], and neurological disorders [51].

Self-efficacy is defined as the perceived capability to perform specific actions required to achieve concrete goals. In individuals with chronic disease, self-efficacy is defined as the patient’s perception of their ability to manage various aspects of their illness and its treatment [52]. It will be assessed using the 4-item General Self-Efficacy Questionnaire. Each item is rated on a scale from 1 to 10, where 0 indicates a lack of confidence in managing the indicated actions, and 10 represents complete confidence in handling the specified situation. Higher scores reflect greater self-efficacy. This instrument has demonstrated strong reliability and validity and has been widely validated in various clinical populations. The Spanish version demonstrates high internal consistency and test–retest reliability, with a Cronbach’s alpha coefficient ranging from 0.88 to 0.95 [53]. Patient adherence [54] and satisfaction with the intervention will also be assessed. To this end, an adherence diary will be used [55], along with a satisfaction questionnaire [56].

In addition to adherence and satisfaction, feasibility will be assessed to evaluate the practicality of implementing the telematic home-based programme. Feasibility outcomes will include: (1) the number and type of technical issues encountered during supervised online sessions; (2) the proportion of successfully completed telematic sessions; (3) participant-reported ease of use and accessibility of the video-conferencing platform; and (4) a brief self-reported questionnaire evaluating participants’ baseline digital literacy and access to compatible devices (smartphone, tablet, or computer). These data will be analyzed descriptively and will provide valuable information on the acceptability and technical viability of telematic interventions in patients with permanent colostomies.

#### 2.7.2. Covariables

Anthropometric data such as age, gender, and body mass index, and clinical data such as personal medical history, indication for surgery, regular medication use and comorbidities assessed with the Charlson Comorbidity Index will be recorded [37].

### 2.8. Sample Size Calculation

An a priori sample size calculation was based on the minimum clinically important difference (MCID) of the physical activity level interference estimated to be 1100 daily steps [57]. Using G*Power version 3.1.9.2, we obtained a sample size of 44 participants, equally distributed between the treatment and control groups, based on a confidence level of 95% (α = 0.05) and a power of 80% (β = 0.2). Anticipating a 15% dropout rate, the total sample size was increased to ensure adequate statistical power, resulting in a sample size of 51 participants.

### 2.9. Statistical Analysis

Data will be analyzed using the Statistical Package for the Social Sciences (SPSS) for Windows (version 26; IBM, Armonk, NY, USA). Categorical data will be expressed as frequency (percentage). Continuous variables will be presented as mean ± standard deviation (SD). The normality of the data will be tested using the Shapiro–Wilk test. Effect sizes will be reported using partial eta-squared (η^2^) and interpreted according to standard conventions.

To evaluate the effects of the intervention over time, a Mixed ANOVA with a Group (intervention vs. control) × Time (baseline, post-intervention, 6 months) interaction will be conducted for all primary outcomes. Post hoc comparisons with Bonferroni correction will be performed if significant interactions are found. Secondary analyses will include intention-to-treat and per-protocol approaches as described above. Statistical significance will be established at *p* < 0.05.

## 3. Results

The results will be published as a peer-reviewed article. The authors intend to include three tables summarizing the main results of the study. The first will present the descriptive characteristics of the sample. The second will report the baseline group comparisons for the primary outcomes. The third table will display the post-intervention results, including both within-group and between-group changes, and the effect sizes will be calculated using Cohen’s d. Additionally, a figure depicting the study flow diagram will be provided.

## 4. Discussion

This study is expected to demonstrate the effectiveness of the intervention in enhancing physical activity and quality of life compared with the control group, while also reducing kinesiophobia and improving exercise capacity and self-efficacy. The results of this study are expected to demonstrate the effectiveness of the intervention in enhancing physical activity and quality of life compared with the control group. Moreover, the programme is expected to reduce kinesiophobia and enhance both exercise capacity and self-efficacy. Through this study, the authors aim to provide clinicians with an evidence-based intervention capable of addressing both the physical and psychological challenges inherent to postoperative recovery.

The study sample is expected to be heterogeneous, as permanent colostomy is performed for a range of underlying conditions. Considerable variability is also anticipated in anthropometric characteristics. For instance, patients with Crohn’s disease who undergo permanent colostomy typically receive this treatment in the third or fourth decade of life [58], whereas those with colorectal cancer generally undergo surgery between 65 and 70 years of age [59,60,61]. Sex-specific variability is also expected: colorectal cancer is more prevalent among men [59,62], while colostomies performed for conditions such as diverticulitis are more common among women [63,64]. This heterogeneity will need to be considered when interpreting the results, as baseline differences in functional capacity, lifestyle habits, and comorbidities may influence the magnitude of the intervention’s effects. A carefully planned randomization process should help balance these factors between groups, but subgroup analyses may also be required to better understand whether certain patient profiles will benefit more from the programme than others.

Extensive scientific literature assesses the quality of life in patients with ostomies, consistently showing that their quality-of-life scores are lower compared to non-ostomized controls [8,9,15,21,43,61,65,66,67]. It is well established that these individuals experience multiple impairments across physical, psychological and social domains [6,68]. By incorporating this variable, the authors seek to corroborate the findings of previous studies and to explore the potential correlation with other variables encompassed in the study. Particularly, it is plausible that improvements in physical activity and exercise capacity mediated by the intervention may indirectly enhance social participation and body image, leading to better overall quality of life. Identifying such relationships could be highly relevant for clinicians, as it would support the integration of physical rehabilitation programmes as part of holistic postoperative care.

Previous research has assessed physical activity levels in ostomized patients using both questionnaires and pedometers [16,69,70]. Overall, these studies indicate that most patients experience a reduction in physical activity following surgery and encounter multiple physical, psychological and social barriers that hinder their return to exercise [17]. These findings underscore the need for structured interventions to facilitate reintegration into active lifestyles. However, the available evidence is highly heterogeneous, as many studies have included mixed samples of patients with ileostomies and colostomies, often performed for oncological indications [20,69,70]. In this context, our study will be novel in focusing exclusively on individuals with permanent colostomies resulting from diverse conditions, thereby providing a more specific perspective on this patient population. By narrowing the scope to this subgroup, the study is expected to generate more precise and clinically meaningful conclusions, overcoming one of the main limitations of previous investigations.

Most investigations on quality of life in patients with permanent colostomies have focused on variables such as anxiety, depression, body image and overall quality of life [14,24,71,72,73]. To date, no studies have specifically aimed to investigate kinesiophobia or fear of movement in this population. This represents an additional strength of the study, and it will generate novel and clinically relevant information that may be associated with decreased physical activity levels and exercise-related barriers. This variable has been extensively studied in other populations such as low back pain [74], knee osteoarthritis [75], chronic pain [30], neck pain [76] and chronic musculoskeletal pain [77,78,79,80]. Similar findings have been reported in oncological rehabilitation [81,82,83,84], where kinesiophobia has been identified as a major barrier to functional recovery and reintegration into physical activity. These parallels support the relevance of addressing fear of movement as a key therapeutic target in patients living with a permanent colostomy. Extrapolating from these populations, it is reasonable to hypothesize that kinesiophobia could represent a central barrier to recovery in colostomy patients, acting as a mediator between physical impairments and reduced participation in daily activities. If the programme proves effective in reducing kinesiophobia, it could open new avenues for integrating cognitive-behavioural principles into physiotherapy-based interventions, fostering a multidisciplinary approach.

Assessment of physical capacity in patients who have undergone permanent colostomy is essential to ensure appropriate progression during exercise programmes. The Six-Minute Walk Test (6MWT) has been widely used in the rehabilitation of patients undergoing colorectal surgery [85] and other populations such as heart failure [86] and COPD [87]. However, there is limited scientific evidence regarding the use of this measure in patients with colostomies [88]. Therefore, the study will provide valuable insight into the six-minute walk test as an objective measure of physical capacity before and after treatment. The inclusion of this functional test will be relevant not only for research purposes but also for practical applications, allowing clinicians to adopt a simple and reproducible tool to monitor progress in real-world settings.

Self-efficacy is a widely studied variable in patients living with a permanent colostomy [68]. Previous studies have reported a direct association between self-efficacy and self-esteem [89]. Social support has also been identified as a key determinant of higher self-efficacy [90,91]. Lower levels of self-efficacy following surgery have been linked to more long-term psychosocial difficulties and worse adaptation to life with a colostomy [92]. Although several programmes have aimed to improve self-efficacy, most have primarily targeted psychological components [91]. To date, there is limited evidence regarding the role of exercise programmes in enhancing self-efficacy. The present study will therefore investigate whether postoperative exercise interventions can strengthen this capacity. If confirmed, these results could support a paradigm shift in which rehabilitation after colostomy is not only conceived as a way to restore mobility but also as a strategy to empower patients, improve their self-management skills, and ultimately promote autonomy and resilience in daily life.

Despite the strengths of the proposed study, several challenges may arise, particularly in patient recruitment and adherence to the intervention. Individuals living with a colostomy often experience diverse biopsychosocial impairments that could limit their participation in exercise programmes. In addition, the sample is expected to be heterogeneous, as patients undergoing this surgery vary widely in age, underlying condition, and comorbidities. This variability may influence baseline functional capacity and the magnitude of treatment effects. To mitigate these factors, the programme has been designed with individualized exercise progression and continuous monitoring, and a stratified analysis will be performed to explore potential subgroup differences.

Ensuring adherence in long-term, home-based interventions is also challenging, as motivation is strongly influenced by perceived benefits and barriers. To enhance engagement, participants will receive clear explanations of all study phases and continuous educational support. The intervention will further incorporate weekly telematic supervision, motivational reinforcement, and individualized feedback from the physiotherapist. These strategies are expected to optimize adherence, reduce dropout rates, and improve the overall feasibility and clinical applicability of the programme.

The methodological strengths of this trial include the detailed design of the intervention protocol, which enhances reproducibility in clinical practice. The individualized progression of graded exposure exercises, tailored to the physical limitations and improvements of each participant, constitutes a distinctive feature. Furthermore, random allocation of participants and blinding of the research assistant to both the study hypothesis and group assignment increase the internal validity of the study design. Another important strength is the pragmatic nature of the intervention: by being implemented in patients’ own homes, it reflects real-life conditions, increasing the potential for scalability and integration into standard care pathways if found effective.

Moreover, the telehealth-based delivery of the programme represents an essential component of its pragmatic design. Remote supervision and digital communication allow continuous professional monitoring without requiring patients to travel, which may reduce dropout rates and improve adherence—particularly among individuals with mobility limitations or those living in rural areas. The integration of telehealth into postoperative rehabilitation also facilitates real-time feedback, individualized progression, and early identification of potential complications, thereby enhancing both safety and engagement. From a public health perspective, this telematic framework offers a scalable and cost-efficient model that could be adapted to different healthcare systems and extended to other patient populations requiring long-term follow-up and support.

## 5. Conclusions

In conclusion, the graded home-based exercise programme is expected to improve physical activity, quality of life, kinesiophobia, exercise capacity, and self-efficacy in individuals with permanent colostomies, providing evidence to support the individualized prescription of exercise in clinical practice. The expected outcomes will provide evidence that such a programme enhances not only physical health but also psychological wellbeing by reducing kinesiophobia and strengthening self-efficacy. Despite potential limitations, this research will provide a foundation for the individualized prescription of exercise in patients living with permanent colostomies.

## Data Availability

This article is a study protocol; therefore, no research data have yet been collected or generated. For this reason, there are no datasets to be shared at this stage. Once the study is completed, data will be available upon reasonable request in accordance with ethical and institutional regulations.
